# Pharmacokinetics of mefloquine and its effect on sulfamethoxazole and trimethoprim steady-state blood levels in intermittent preventive treatment (IPTp) of pregnant HIV-infected women in Kenya

**DOI:** 10.1186/s12936-015-1049-9

**Published:** 2016-01-05

**Authors:** Michael Green, Kephas Otieno, Abraham Katana, Laurence Slutsker, Simon Kariuki, Peter Ouma, Raquel González, Clara Menendez, Feiko ter Kuile, Meghna Desai

**Affiliations:** Division of Parasitic Diseases and Malaria, Centers for Disease Control and Prevention, Center for Global Health, Atlanta, GA USA; Kenya Medical Research Institute, Centre for Global Health Research, Kisumu, Kenya; ISGlobal, Barcelona Ctr Int Health Res. (CRESIB), Hospital Clínic, Universitat de Barcelona, Barcelona, Spain; Liverpool School of Tropical Medicine, Liverpool, UK

**Keywords:** Malaria, Pharmacokinetics, Mefloquine, Sulfamethoxazole, Trimethoprim, Pregnant, HIV

## Abstract

**Background:**

Intermittent preventive treatment in pregnancy with sulfadoxine/pyrimethamine is contra-indicated in HIV-positive pregnant women receiving sulfamethoxazole/trimethoprim prophylaxis. Since mefloquine is being considered as a replacement for sulfadoxine/pyrimethamine in this vulnerable population, an investigation on the pharmacokinetic interactions of mefloquine, sulfamethoxazole and trimethoprim in pregnant, HIV-infected women was performed.

**Methods:**

A double-blinded, placebo-controlled study was conducted with 124 HIV-infected, pregnant women on a standard regimen of sulfamethoxazole/trimethoprim prophylaxis. Seventy-two subjects received three doses of mefloquine (15 mg/kg) at monthly intervals. Dried blood spots were collected from both placebo and mefloquine arms four to 672 h post-administration and on day 7 following a second monthly dose of mefloquine. A novel high-performance liquid chromatographic method was developed to simultaneously measure mefloquine, sulfamethoxazole and trimethoprim from each blood spot. Non-compartmental methods using a naïve-pooled data approach were used to determine mefloquine pharmacokinetic parameters.

**Results:**

Sulfamethoxazole/trimethoprim prophylaxis did not noticeably influence mefloquine pharmacokinetics relative to reported values. The mefloquine half-life, observed clearance (CL/*f*), and area-under-the-curve (AUC_0→∞_) were 12.0 days, 0.035 l/h/kg and 431 µg-h/ml, respectively. Although trimethoprim steady-state levels were not significantly different between arms, sulfamethoxazole levels showed a significant 53 % decrease after mefloquine administration relative to the placebo group and returning to pre-dose levels at 28 days.

**Conclusions:**

Although a transient decrease in sulfamethoxazole levels was observed, there was no change in hospital admissions due to secondary bacterial infections, implying that mefloquine may have provided antimicrobial protection.

## Background

In many parts of Africa, pregnancy is complicated by co-infection with malaria and human immunodeficiency virus (HIV). Maternal malaria was associated with a two-fold higher HIV-1 viral concentration, and in areas where HIV prevalence is greater than 10 % of the population, the proportional increase of malaria during pregnancy can be from 5.5 to 18.8 % [1]. Some reports show that malaria increases the incidence of mother-to-child HIV transmission as well as contributing to low birth weight and maternal anaemia [1, 2]. In addition, drugs used to treat HIV and associated infections may cause serious drug interactions. For example, HIV-infected patients receiving co-trimoxazole (sulfamethoxazole/trimethoprim) prophylaxis should not take another sulfonamide-containing drug, such as sulfadoxine/pyrimethamine (SP), as the risk of sulfonamide-induced adverse effects is increased. Since physiological functions are altered during pregnancy, the pharmacokinetics of many anti-malarials are different in pregnant women compared to non-pregnant women [3–5].

The only drug currently recommended for malaria intermittent preventive treatment (IPTp) during pregnancy is SP [6]. The development of resistance to SP, as well as safety concerns, has limited the options for malaria prevention in pregnant women [7–9]. The Global Malaria-in-Pregnancy Consortium aims to identify alternatives to SP for IPTp, and has considered mefloquine (MQ) as a potential replacement for SP-IPTp. Although MQ has been used for treatment of multidrug-resistant *Plasmodium falciparum* infections since the 1960s, there have been few MQ pharmacokinetic studies involving pregnant women. Pharmacokinetic parameters of many drugs are altered in pregnant women due to the differences in the physiological functions relative to non-pregnant women. For example, a controlled study of pregnant and non-pregnant women infected with *P. falciparum* showed a significantly lower maximum blood concentration (C_max_) and a larger volume of distribution (Vd/F) of MQ in the pregnant group [10]. In addition, interactions with other medications commonly used as prophylaxis against opportunistic infections in HIV-infected individuals may influence MQ pharmacokinetics.

The effects of various drugs on co-trimoxazole (CTX) levels and the effects of CTX on certain drug levels have been assessed in numerous studies. Co-administration of lamivudine with CTX resulted in an increased drug exposure and decreased clearance of lamivudine [11] while disposition of indinavir [12], maraviroc [13] or zidovudine [14] was not affected by co-administration with CTX. Rifampicin was shown to reduce both trimethoprim and sulfamethoxazole concentrations in HIV-infected patients [15]. To date, there are no data on the interaction between CTX and MQ.

A multicentre, randomized, placebo-controlled, clinical trial was conducted to evaluate the efficacy of MQ for IPTp among HIV-infected women receiving CTX (NCT 00811421: Pan African Clinical Trials Registry PACTR 2010020001813440) [16]. As part of this larger trial, a nested investigation of the pharmacokinetics of MQ and its effect on the blood levels of CTX was conducted in a sub-sample of HIV-infected pregnant women enrolled into the main trial at the site in Kisumu, Kenya.

## Methods

### Subjects

The double-blinded, placebo-controlled study took place at the Siaya District Hospital, situated in an area of western Kenya with holo-endemic malaria transmission and a high prevalence of HIV. The main trial has been described in more detail elsewhere [17]. Following clinical examination, women with a gestational age ≤28 weeks were included in the study, whereas IPTp was given only if the gestational age was greater than 13 weeks. The MQ arm received the first dose of IPTp (15 mg/kg MQ) under supervision along with a continued daily dose of CTX (800 mg sulfamethoxazole, 160 mg trimethoprim). Each MQ tablet contains 250 mg of mefloquine HCl. The number of MQ tablets administered to a woman was determined according to the maternal weight at the time of first IPTp administration (i.e., a woman weighing 60 kg received three-and-a-half MQ tablets). Women in the placebo arm received placebo MQ at the same schedule as for the MQ arm, as well as CTX. CTX was administered daily and a total of three doses of MQ or placebo were administered at least 1 month apart. HIV-infected pregnant women who met all the enrolment criteria for the main trial, and reported having taken CTX on a daily basis in the previous 7 days, were invited to participate in this nested study. Participants from the main study who verbally reported having taken CTX on a daily basis in the past week underwent a standard informed consent procedure at enrolment into the nested pharmacokinetic (PK) study. The institutional review boards of the Kenya Medical Research Institute (Nairobi, Kenya), the US Centers for Disease Control and Prevention (Atlanta, GA, USA) and the Hospital Clinic of Barcelona (Spain) approved the study protocol. All women who participated gave written informed consent.

### Sampling frame

A total of 124 HIV-infected pregnant women were selected for MQ, sulfamethoxazole (SMX) and trimethoprim (TMP) analysis. After disclosure of the MQ and placebo groups, 72 of 124 subjects had received MQ. The PK parameters for MQ were determined from this group. The subjects were randomly divided into three groups: Group A provided blood samples at two, 14 and 28 days, Group B provided samples at 4 h, 1 and 7 days, and Group C provided samples at 8 h, 3 and 21 days. On days 0, 1, 3, 7, 14, 21, and 28, blood samples were collected prior to the next dose of co-trimoxazole in order to obtain the minimal steady-state concentrations of SMX and TMP. All the subjects provided a sample at day 0 (pre-dose). Final number of samples collected per time point ranged from 13 to 22 with a median of 18. Sixteen samples per time point is enough to detect 25 % change (α = 0.05, β = 0.02), considering the estimated % coefficient of variation (% CV) for inter-individual MQ blood concentration to be 35 % (derived from previous laboratory experience performing MQ analyses) (MedCalc Statistical Software version 13.2.0 (MedCalc Software bvba, Ostend, Belgium). At day 7 following a second monthly dose of MQ, blood was collected for analysis to observe any accumulation.

### Blood sample collection

At the appropriate time point, approximately 0.5 ml whole blood was collected by venipuncture. Three blood spots were prepared on Whatman No 1 filter paper by transferring 0.1 ml of whole blood using a positive displacement pipette. The spotted filter papers were air-dried, individually wrapped, labelled, and transferred to plastic bags containing a small amount of silica to prevent moisture accumulation. All dried blood spots were stored in a refrigerator (4 °C) until transported to CDC via travelling personnel. After delivery, samples were stored at −20 °C until analysis.

### Analytical procedure

A modification of a high-performance liquid chromatographic (HPLC) method described by Bergqvist et al. was used to simultaneously measure TMP, SMX, and MQ in dried blood spots [18]. TMP, SMX and MQ analytical standards were generously donated by WWARN (Worldwide Antimalarial Resistance Network). A 4.6 × 150 mm, 5 μ, C18 column (Supelco) coupled to a 2.1 × 10 mm, 3.5 μ, RP8 guard column (Symmetryshield) and a mobile phase consisting of 30 % acetonitrile and 70 % water containing 20 mM *N*,*N*-dimethyloctylamine adjusted to pH 2.3 with phosphoric acid was used to separate the components. The column temperature was set for 30 °C and the mobile phase flow rate set for 1 ml/min. The components were detected using a diode array detector with wavelength set at 280 and 220 nm. Drug extraction from dried blood spots was accomplished by combining a solid phase technique for sulfonamides [19] with liquid–liquid extraction for MQ [18]. Samples were analysed in duplicate. The dried blood spots were cut into four sections and inserted into a polypropylene tube containing 100 ml of phenacetin (10 μg/ml in methanol) as an internal standard for SMX and TMP quantitation and 50 ml of the mefloquine derivative WR184806 (29 mg/ml in methanol) as an internal standard for MQ quantitation; 1.5 ml of sodium hydroxide (0.1 M) was added and the papers allowed to soak for 10 min. The tubes were rotated for 15 min followed by removal of the filter papers. A volume of 0.25 ml zinc sulfate (1 M) was added and the tubes rotated for 15 min followed by centrifugation for 10 min at 4000 rpm. The supernatant was added to prepped 100 mg C8 solid-phase extraction columns and eluted by vacuum. The column matrix was washed with 0.5 ml of water and the SMX and TMP eluted with 1 ml of acetone containing 2 % triethylamine. For the MQ extraction, 1.5 ml of pH 9 phosphate buffer was added to the pellet and sonicated for 10 min; 5 ml of methyl *t*-butyl ether was added and the tubes rotated for 10 min followed by centrifugation for 5 min at 2000 rpm. The organic phase layer was transferred to the appropriate tubes containing the acetone eluent. Extraction with methyl *t*-butyl ether was repeated and the organic phase combined with the previous extracts from the appropriate samples. The extract was evaporated to dryness with nitrogen, reconstituted in mobile phase and injected into the HPLC for analysis. The extraction method was not optimized for measuring blood levels of the major metabolites, carboxymefloquine and *N*-acetyl sulfadoxine, although these components were effectively separated by HPLC. Blood concentrations for each component were determined from a standard curve consisting of a series of spiked dried blood spots. Evaluation of the analysis method for ten standard curve runs showed the % coefficient of variation to be 11, 7 and 26 % for MQ (594 ng/ml), SMX (27.5 mg/ml) and TMP (1.8 mg/ml), respectively. The relatively high variation for TMP was a result of the low concentrations in blood which was approaching detection limits. The % accuracy was 4.2, 1.5 and 0.2 % for MQ, SMX and TMP, respectively. Some samples had large unidentified chromatographic peaks that interfered with the analytes, possibly resulting from other ingested medications. Therefore, blood concentration values for each time point were subjected to the Tukey Outlier Test [20] where values larger than the upper quartile plus three times the interquartile range were eliminated. Only one outlier, occurring at day 7 was eliminated from the MQ concentration–time curve. No more than two outliers per time point was eliminated from the TMP data. The remaining blood concentration values were then averaged for each time point and used for the pharmacokinetic analysis.

### Pharmacokinetic analysis

**Fig. 1:**
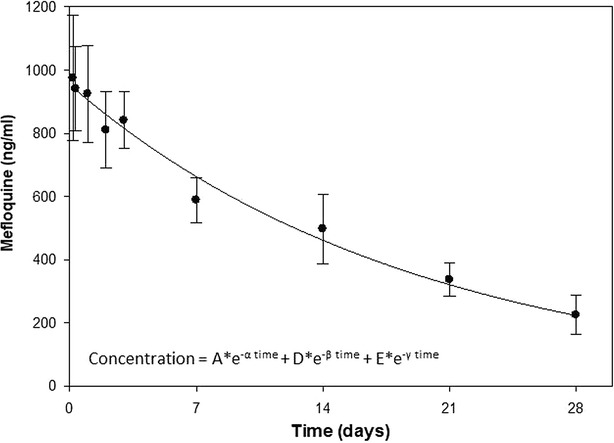
Mefloquine concentration (average ± SE) versus time profile

**Fig. 2:**
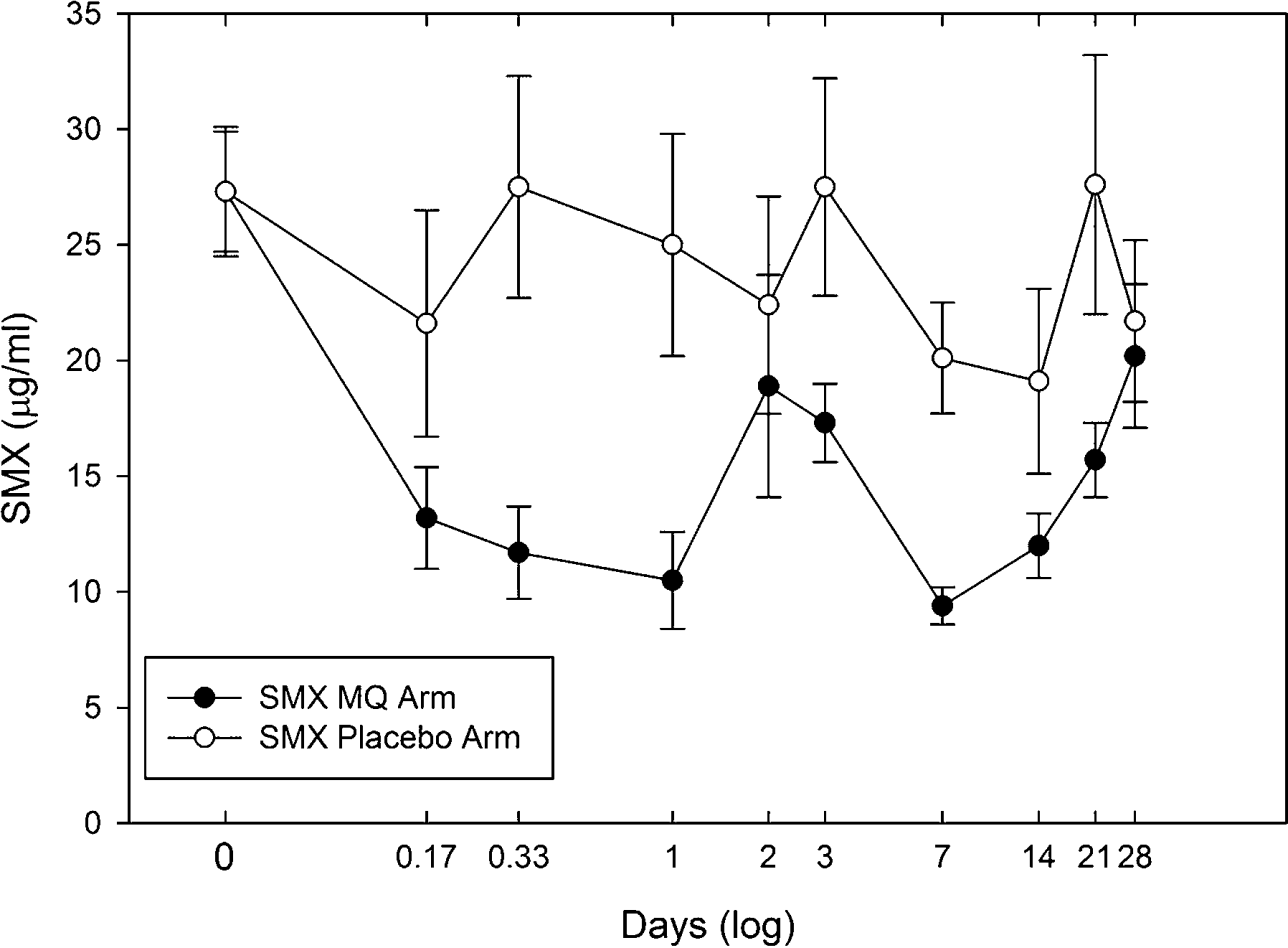
Comparison of sulfamethoxazole blood levels (average ± SE) by treatment group

Pharmacokinetic parameters for MQ were calculated based on the use of non-compartmental methods of analysis of naïve-pooled data (NPD) using PK Solutions Vers. 2.0 Pharmacokinetic Data Analysis software (Summit Research Services Montrose, CO, USA). The software uses a method based on curve stripping, which resolves the concentration–time curve into a series of exponential terms used to calculate the various PK parameters. A tri-exponential equation (Concentration = A × e^−α time^ + D × e^−β time^ + E × e^−γ time^), previously used to describe MQ pharmacokinetics [21], was initially applied to create the MQ concentration–time profile (Fig. 1) and provide test values for the curve stripping process. This equation focuses primarily on the distribution and elimination phases of the pharmacokinetic curve and is appropriate in this circumstance where absorption phase kinetics is not truly represented due to the initial sample collection at 4 h. Linearity in the terminal portion of a semi-log plot confirmed apparent first-order rate processes. Blood samples taken at days 0, 1, 3, 7, 14, 21, and 28 prior to the next dose of co-trimoxazole represented the minimal concentration [C_ss_ (min)] that occurs during steady state. Therefore, the averages of these values were used to determine the steady-state [C_ss_ (min)] values for TMP and SMX (Fig. 2). Analysis of variance (ANOVA) was performed on the TMP and SMX concentration–time data. The rate at which SMX was increasing was determined by using an exponential growth equation (y = ae^bx^), where ‘b’ is the rate constant.

## Results

### Mefloquine pharmacokinetics

Although the population pharmacokinetics (popPK) approach can accommodate sparse data and provide information on the impact of covariates, it requires the use of sophisticated software, which may not be available to everyone. A comparison of the popPK approach with NPD offers no real advantages when determining PK parameters from small sample sizes [22]. Therefore, the NPD approach was used. The characteristics of the study participants are shown in Table 1. The PK parameters for MQ, obtained from the concentration–time profile shown in Fig. 1 are compared with parameters from similar studies (Table 2). A recent study: Rich and population pharmacokinetics of mefloquine intermittent preventive treatment for malaria in pregnancy in Gabon (Ramharter et al. pers comm) was also conducted as part of a multicentre trial (NCT 00811421) with parameters included in Table 2. Relative to the area-under-the-curves (AUCs) from the Gabon trial and Na Bangchang et al. [10], the data suggest HIV-infected pregnant women taking CTX may result in enhanced MQ bio-availability. Future studies using a population-based analysis would be better able to determine which parameter is responsible for the suggested interaction. The terminal elimination half-life (t_1/2_ = 12.0 days) was similar to the mean half-life of 11.6 days for whole blood MQ in pregnant women given 125 or 250 mg per week during the third trimester of pregnancy [9]. *Plasmodium falciparum*-infected pregnant and non-pregnant Thai women showed more rapid whole blood MQ half-lives of 7.2 and 8.1 days, respectively [10]. In comparison, the half-life for whole blood MQ in healthy individuals was reported to be 13.8 days [21], while plasma half-lives range from 16 to 28 days [23–29]. The systemic clearance (CL/*f*) determined from this study was 0.035 l/h/kg. The AUC_0→∞_ of 431 µg-h/ml determined from the study was higher than that reported from healthy pregnant women (~300 µg-h/ml) and pregnant women infected with *P. falciparum* (319 µg-h/ml) [9, 10]. The observed t_max_ and C_max_ were 4 h and 974 ng/ml and within the range of values reported from normal pregnant women (t_max_ = 6, range 3–34 h) and *P. falciparum* pregnant women given 15 mg/kg of mefloquine (C_max_ = 1257, range 650–1584 ng/ml) [9, 10]. The t_max_ and C_max_ determinations are heavily biased since the first sample was collected at 4 h and presents a limitation of the study design. The MQ levels at 7 days post second dose of 594 ± 80 ng/ml (mean ± SE, n = 16) was not significantly different (p = 0.4) from levels at 7 days post first dose (490 ± 99 ng/ml, n = 11), indicating no significant drug accumulation. Although at three half-lives, some accumulation can be expected, large inter-individual variability as well as a small sample size may account for the lack of significant drug accumulation.

**Table 1:** Characteristics of subjects upon enrolment

	MQ arm (n = 72)	Placebo arm (n = 52)
Age (years)	27 (17–42)	27 (17–42)
Body weight (kg)	57 (40–77)	60 (42–80)
Gestation (weeks)	20 (8–28)	20 (8–28)
Haemoglobin (mg/dL)	10.3 (5.6–15.2)	10.6 (5.7–13.7)

Median (range)

**Table 2:** Comparison of pharmacokinetic parameters for mefloquine

Reference	Matrix	Subject description	Dose (mg)	C_max_ (ng/ml)	t_1/2_ (days)	Vd (l/kg)	CL (l/h/kg)	AUC_0→∞_ (µg-h/ml)
Present study	Whole blood	Pregnant	15 mg/kg	974	12.0	14.4	0.035	431
Ramharter et al. (pers comm)	Whole blood	Pregnant	15 mg/kg	577^a^	16.8	30.4^b^	0.073^b^	308^a^
Desjardins et al. [21]	Whole blood	Males	1000	800	13.8	13.3	0.066	648
Schwartz et al. [23]	Plasma	African males	1000	954	20.0	14.8	0.020	–
		Caucasian males	1000	990	27.5	20.3	0.025	
De Souza et al. [26]	Plasma	Males	1000		21.6	23.2	0.031	587
Na Bangchang et al. [10]	Whole blood	Pregnant (*P. falciparum*)	15 mg/kg	1257	7.2	10.8	0.047	319
	Whole blood	Non-pregnant Females (*P. falciparum*)	15 mg/kg	1617	8.1	10.0	0.043	349
Nosten et al. [9]	Whole blood	Pregnant	250 mg/week	–	11.6	–	0.047	–
			125 mg/week					

^a^Original values converted from moles to grams using the molar mass for mefloquine HCl (414.8 g/mole) for comparison

^b^Original values divided by mean weight (58.3 kg) of the study subjects for comparison

### Sulfamethoxazole/trimethoprim

**Fig. 3:**
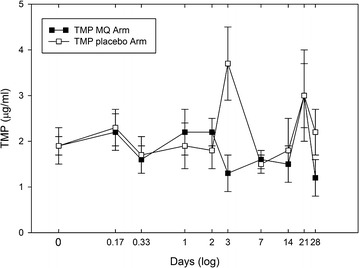
Comparison of trimethoprim blood levels (average ± SE) by treatment group

The presence of MQ did not significantly affect the steady-state minimum concentrations [C_ss_ (min)] for TMP (2.23 µg/ml in placebo arm vs 1.86 µg/ml in MQ arm) (Fig. 3). Although the analytical precision (% CV = 26) for TMP measurements may have masked any significant differences between arms, these concentrations are consistent with those found in healthy adults given the same dose (Table 3) [30–32]. In contrast, the [C_ss_ (min)] for SMX showed as much as a 53 % decrease in the MQ arm relative to the placebo arm. An ANOVA analysis reveals significant changes in the SMX concentration–time profile in the MQ arm (p < 0.0002) while no significant changes (p > 0.05) were observed for SMX in the placebo arm. It was observed that as MQ is being eliminated, SMX concentrations gradually increase back to original levels (Fig. 2). The average [C_ss_ (min)] for SMX in the placebo arm (23.8 µg/ml) is less than that reported in healthy adults taking the same dose (Table 3) [30–32]. Since HIV status has been shown to have no effect on the pharmacokinetics of either SMX or TMP [33], it is conceivable that low absorption or faster metabolism of SMX during pregnancy may occur. In a study of the pharmacokinetics of sulfadoxine–pyrimethamine in HIV-infected pregnant women, it was observed that pregnancy contributes to lower AUC and shorter half-life for sulfadoxine, while HIV status did not show any significant influence [34].

**Table 3:** Reported minimum steady-state values C_ss_ (min) for sulfamethoxazole (SMX) and trimethoprim (TMP) in previous studies

SMX (mg ml^−1^)	TMP (mg ml^−1^)	References
23.8	2.2	Present study
45.5^a^	2.2^a^	Kremers et al. [32]
35.6	1.5	Nowak et al. [31]
43.4	2.98	Kaplan et al. [30]

^a^Plasma concentration converted to whole blood equivalents using blood/plasma ratio [39]

Drug interactions can occur as a result of competitive or non-competitive inhibition of the Cytochrome p450 enzyme. The Cytochrome p450 isoform, CYP3A4, has been implicated in MQ metabolism since the co-administration of ketoconazole, a potent inhibitor of CYP3A4 was shown to significantly increase plasma MQ levels in humans [35]. Since SMX and TMP have negligible effects on the CYP3A4 isoform [36], it is expected that SMX and TMP will have little effect on MQ pharmacokinetics. Although, the results did not show any significant changes in MQ pharmacokinetics in the presence of steady-state levels of SMX and TMP, a significant reduction of SMX levels upon MQ administration was observed. A reduction of SMX levels has also been observed in HIV-infected individuals administered rifampin and has been attributed to enhanced SMX hepatic acetylation [15]. MQ or its metabolite, carboxymefloquine may affect SMX metabolism similarly. The rapid reduction of SMX levels 4 h after MQ administration (Fig. 2) may be attributed to the presence of either MQ or its metabolite, carboxymefloquine. If MQ has a direct effect on SMX metabolism, SMX levels would be expected to rise after MQ begins its elimination phase at approximately 4 h. Although an unexplained elevation of SMX levels is seen at day 2, the lowest levels occur at day 7 followed by an exponential rise to MQ pre-dose levels at day 28. The rise in SMX levels at day 7 corresponds to the beginning of the elimination phase (t_max_ = 9 ± 3 days) for the carboxymefloquine metabolite [35]. The rate of SMX increase was determined by fitting an exponential growth equation (y = ae^bx^) to SMX concentrations from seven to 28 days. The growth constant (b) of 0.0015 h^−1^ is equal to a doubling time of 19 days. The half-life for carboxymefloquine of 21 days [35] corresponds to the doubling time of SMX of 19 days, suggesting that the presence of the carboxymefloquine metabolite may influence SMX metabolism. A recent study has shown that the major metabolite of MQ, carboxymefloquine induces the expression of drug metabolizing enzymes and transporters by activating the pregnane X receptor [37].

Limitations of this study include the lack of sufficient blood samples collected during the MQ absorption phase to obtain accurate t_max_, C_max_, as well as the absorption rate constant (k_a_). Obtaining multiple blood samples in a short period of time required for absorption phase kinetics is difficult under the conditions encountered in developing countries. Drug metabolites were not quantified using the described analytical technique, therefore any associations with metabolite levels could not be made directly. Although no significant differences were seen for TMP between the MQ and placebo arm, low levels and high variability may have masked any changes. Over 95 % of the subjects in the study were taking an antiretroviral medication (mostly zidovudine, lamivudine, and nevirapine) concomitant with MQ and CTX. Possible interactions with these antiretrovirals cannot be ruled out.

## Conclusions

This is the first study assessing the pharmacokinetic inter-relationship of MQ and CTX. In this study, prophylactic levels of CTX did not significantly alter MQ pharmacokinetics in HIV-infected pregnant women relative to reported parameters in pregnant women not receiving CTX. While exposure to MQ did not affect TMP levels, there is concern that the transient decline in SMX levels would result in an increase in hospital admissions from secondary bacterial infections. However, this was not the case. Results from the multi-site clinical trial from which this study is derived do not support this assertion [17]. This implies that that the antimicrobial properties of MQ may have provided some protection against microbial infections [38].
